# Symmetric sensing and symmetry-breaking processing as a minimal principle for directional inference

**DOI:** 10.1016/j.isci.2026.117184

**Published:** 2026-08-25

**Authors:** Nobuchika Yamaki, Tenna Churiki

**Affiliations:** 1 TNQ Tech, Co., 131 Continental Drive, Suite 305, Newark, DE 19713, USA; 2 King’s College London, London, UK

**Keywords:** bilateral symmetry, lateralization, directional inference, symmetry breaking, sensory processing

## Abstract

Bilateral animals often combine symmetric body plans with lateralized neural processing, but the computational relation between these features remains unclear. We used a minimal two-sensor framework to separate sensing from readout. Across auditory, binocular, and tactile models, symmetric sensor placement produced Z_2_-equivariant Fisher-information profiles, indicating matched local estimation precision for opposite sides of the body midline. Because Fisher information does not directly establish sign recovery, we separately measured total information, sign information, unsigned spatial information, and balanced sign accuracy. In an auditory cross-correlation model, an even scalar readout preserved unsigned source eccentricity but remained nearly sign-blind. Increasing the odd readout component selectively increased sign information, balanced accuracy, correlation magnitude, and odd conditional-mean structure, while reducing unsigned information. A decoder trained on the full cross-correlation also acquired an almost purely odd weight profile. These results show that symmetric sensing balances input precision, whereas antisymmetric readout recovers directional sign.

## Highlights

• Symmetric sensor placement balances local precision across left and right
• Even readouts preserve unsigned spatial information but remain sign-blind
• Odd readout components recover directional sign from symmetric sensory input
• A learned decoder develops an almost purely odd delay-weighting profile

## Graphical abstract

**Figure:**
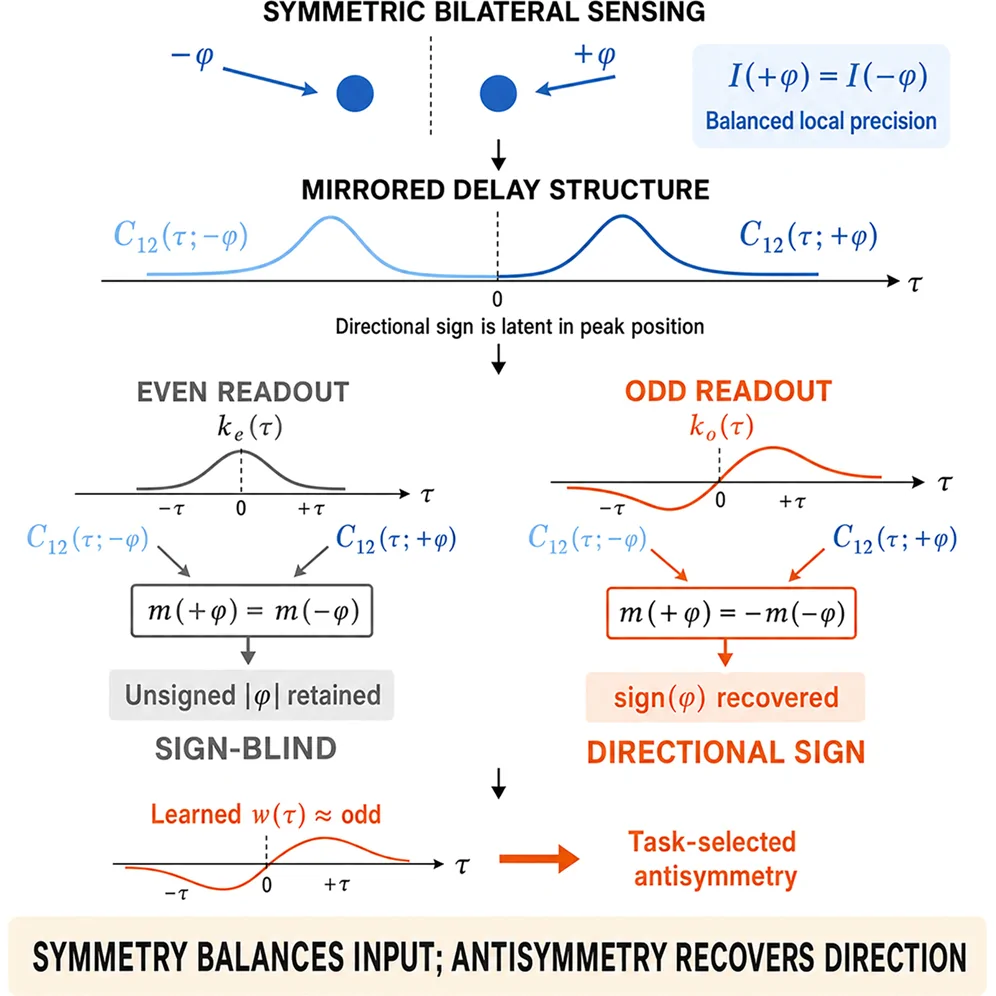

## Teaser

Applied sciences; Sensor system

## Introduction

Bilateral organization is one of the most common structural features of animal bodies. In many motile animals, sensors and effectors are arranged as left-right pairs around a sagittal midline, producing an approximately mirror-symmetric body plan. At the same time, many nervous systems supported by these bodies show functional lateralization, in which perceptual, motor, or cognitive operations are distributed asymmetrically across the two sides. The coexistence of bilateral morphology and lateralized function is well documented across vertebrates and invertebrates, but the computational relation between the two remains insufficiently specified.^1^^,^^2^^,^^3^

Explanations of bilateral symmetry and explanations of neural lateralization often address different biological problems. Bilateral body organization has been linked to directed locomotion, body-axis formation, internal transport, developmental patterning, and biomechanical constraints.^4^^,^^5^ Functional lateralization has instead been discussed in terms of reduced redundancy, parallel specialization, behavioral efficiency, and side-biased neural control.^1^^,^^3^ These explanations are compatible, but they do not by themselves explain why a symmetric body plan and asymmetric processing should frequently coexist within the same organism. A computational account must specify what symmetric sensing contributes and what asymmetric processing adds.

Spatial sensing provides a direct setting in which to formulate this problem. A pair of ears can compare arrival times, a pair of eyes can compare viewing angles, and paired tactile receptors can compare stimulation across the body surface. In each case, the organism may need two different kinds of information. The first is unsigned spatial information: how far a stimulus lies from the body midline. The second is directional sign: whether the stimulus lies on the left or the right side of the midline. A system can, in principle, encode unsigned spatial magnitude without recovering left-right sign. This distinction is essential for understanding why bilateral sensing alone may be insufficient for directional behavior.

The present study formalizes left-right exchange as Z_2_ symmetry. In this biological context, Z_2_ denotes the operation of swapping the left and right sides. For a horizontal source direction φ, this reflection maps φ to −φ. A bilaterally symmetric sensor pair is unchanged by this operation except that the two sensors exchange roles. This provides a precise way to ask whether a sensing stage treats the two sides of the body with equal local precision.

Fisher information was used to characterize this sensing-stage property. In the present framework, Fisher information measures local estimation precision of the sensory cue with respect to the task variable. A Z_2_-equivariant Fisher-information profile means that +φ and −φ are represented with matched local precision. It does not mean that a downstream scalar readout can distinguish left from right. This distinction separates balanced sensing from sign recovery. Balanced sensing concerns the precision with which opposite sides are represented; sign recovery concerns whether a readout variable actually changes with the side of the source.

The readout problem is clearest in delay-based auditory processing. Paired sensor signals can be summarized by their cross-correlation as a function of relative delay τ. A processing kernel is the weighting rule by which this cross-correlation is read. An even kernel treats positive and negative delays equivalently. In biological terms, it does not distinguish which side led in time. An odd kernel assigns opposite signs to opposite delays. In biological terms, it can distinguish left-leading from right-leading structure. Thus, even and odd kernels correspond to different functional operations on the same bilateral input. An even readout can preserve unsigned structure, whereas an odd readout can express directional sign.

Symmetry breaking is also a general concept in physical systems, where symmetric configurations can give rise to asymmetric or antisymmetric states under particular dynamical conditions. Recent studies of nonlinear soliton systems provide examples in which symmetry and antisymmetry breaking arise from the structure of the governing dynamics.^6^^,^^7^ The present work uses symmetry breaking in a more restricted computational sense. The question is whether a bilateral sensory system with symmetric input geometry can recover a signed directional variable without introducing an antisymmetric operation at the readout stage.

The central claim is conditional. Under bilaterally symmetric sensor placement, symmetric source statistics, stationary source signals, independent symmetric noise, and a scalar cross-correlation readout, an even kernel cannot express directional sign through the conditional mean of the readout. It can preserve information about |φ|, but it remains sign-blind. A nonzero odd component supplies the antisymmetric operation required to make the scalar readout sign-sensitive. This is not a claim that all biological directional inference must arise from processing-stage asymmetry. If the sensor geometry itself breaks left-right symmetry, then directional structure can enter at the sensing stage. The claim is that, under symmetric bilateral placement, scalar sign recovery from the cross-correlation requires an antisymmetric readout component.

This formulation provides a computational link between bilateral morphology and functional lateralization. A symmetric sensor arrangement can support left-right balanced local precision, while asymmetric processing can convert the resulting bilateral comparison into a signed directional variable. Bilateral symmetry and lateralized readout are therefore not competing explanations. They perform different operations within the same inference pipeline: the former balances the input stage, and the latter extracts directional sign.

We instantiate this framework in auditory, binocular, and tactile sensing models. These modalities share a common bilateral structure but differ in the physical cue used for spatial inference: interaural time difference, angular disparity, and differential tactile activation.^8^^,^^9^^,^^10^^,^^11^^,^^12^ Across these models, symmetric sensor placement tests whether opposite sides of the task space are represented with matched local precision. The auditory model then provides a direct delay axis for analyzing how even and odd readout components determine whether the paired sensory comparison remains sign-blind or becomes sign-sensitive.

The resulting account is deliberately minimal. It does not attempt to reproduce the full biological architecture of auditory, visual, or tactile circuits. Instead, it isolates a symmetry-defined mechanism that any bilateral system must address when a symmetric input stage is used to support left-right behavior. Symmetric sensing can preserve balanced local precision, but directional sign requires a readout operation that changes sign under left-right reflection.

## Results

### Bilateral sensing and scalar readout were separated into two computational stages

**Figure 1:**
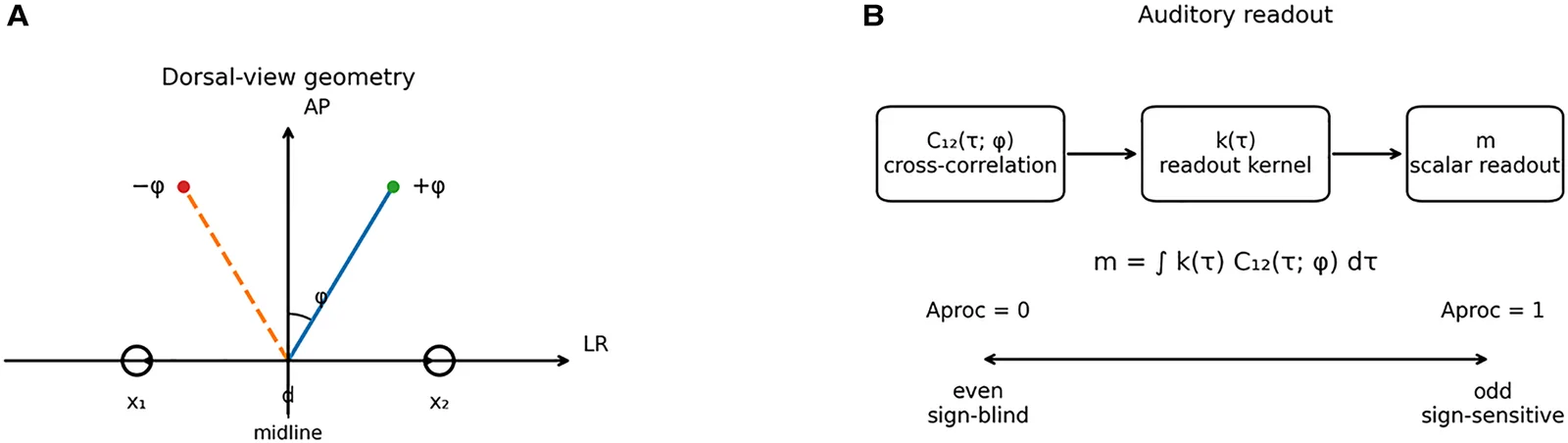
Bilateral two-sensor geometry and readout scheme (A) Minimal bilateral geometry shown in dorsal view. Two sensors, *x*_1_ and *x*_2_, are placed symmetrically about the body midline at separation *d*. The anteroposterior axis is indicated as AP, and the left-right axis is indicated as LR. Source direction is measured by the angle *φ* relative to the AP axis; +*φ* and −*φ* denote mirror-symmetric source directions on opposite sides of the body midline. (B) Auditory processing-stage schematic. The paired sensor signals are summarized by the cross-correlation *C*_12_(*τ*; *φ*), where *τ* is the relative-delay coordinate. A scalar readout is then obtained as *m* = integral of *k(τ)C*_12_(*τ*; *φ*) over *τ*. The parameter *A*proc controls the mixture between an even kernel and an odd kernel, spanning a continuum from purely even processing, *A*proc = 0, to purely odd processing, *A*proc = 1. In the present study, the *τ*-based cross-correlation readout is used only for the auditory processing-stage model.

The model separated bilateral directional inference into a sensing stage and a readout stage. The sensing stage consisted of two sensors placed symmetrically around the body midline in a dorsal-view body plane. In this geometry, the y axis denotes the anteroposterior axis and the x axis denotes the left-right axis. A horizontal source direction φ is measured from the positive anteroposterior axis toward the positive left-right side. Opposite source sides are therefore represented as +*φ* and −*φ*. Under the left-right reflection, +*φ* is mapped to −*φ*, and the two sensors x_1_ and x_2_ are exchanged. This geometry defines the Z_2_ symmetry tested in the sensing-stage analyses (Figure 1A).

The processing stage was analyzed only for the auditory model, where paired sensor signals naturally differ in arrival time. The two auditory signals were summarized by the cross-correlation *C*_12_(*τ*; *φ*), where *τ* is the relative-delay axis between sensors x_1_ and x_2_. Positive and negative values of *τ* correspond to opposite arrival-time orders across the bilateral sensor pair (Figure 1B). The scalar readout m was obtained by weighting *C*_12_(*τ*; *φ*) with a processing kernel *k(τ)*. The processing-asymmetry parameter *Aproc* continuously interpolated between a purely even readout, which treats opposite delay directions equivalently, and a purely odd readout, which assigns opposite signs to opposite delay directions (Figure 1B). Thus, the model tested whether directional sign was available from symmetric sensing alone or required an antisymmetric readout operation.

### Symmetric sensor placement preserved Z_2_ equivariance at the sensing stage

**Figure 2:**
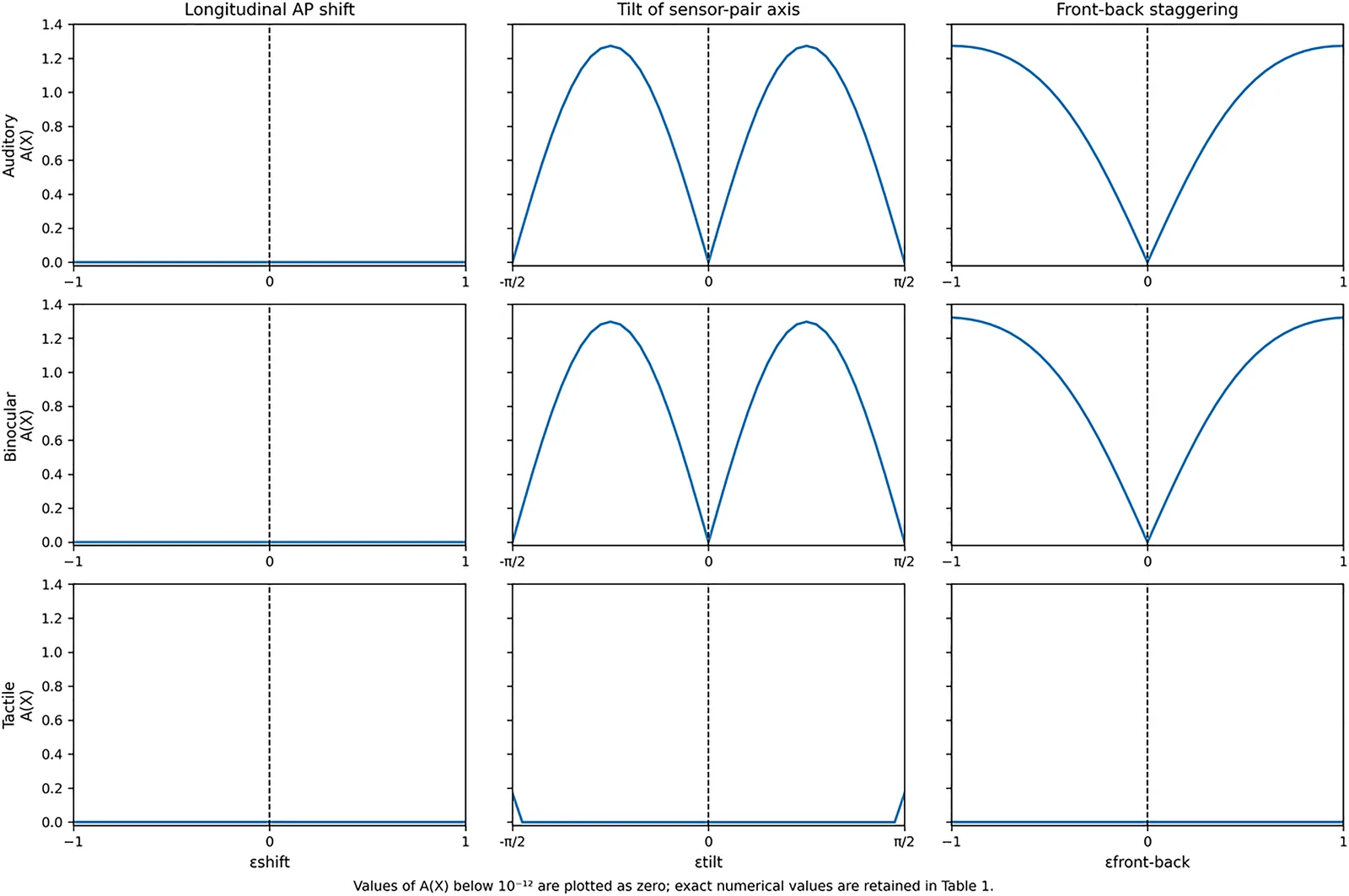
Z_2_ equivariance across modalities under sensor asymmetry Z_2_-violation index *A*(*X*) as a function of three sensor-geometry deformations in auditory, binocular, and tactile sensing. Columns show longitudinal AP shift, tilt of the sensor-pair axis, and front-back staggering. Rows show auditory, binocular, and tactile modalities. The dashed vertical line marks the symmetric configuration. *A*(*X*) = 0 indicates perfect Z_2_ equivariance, whereas larger values indicate stronger violation of mirror symmetry. Auditory and binocular sensing remained Z_2_-equivariant under longitudinal AP shift but showed strong violations under tilt and front-back staggering. Tactile sensing remained at numerical zero across all tested deformations because the tactile cue depended only on the left-right coordinates of the receptors on a one-dimensional body surface. For visual display, values of *A*(*X*) below 10^∧^-12 were plotted as zero; exact numerical values are reported in Table 1.

At the symmetric sensor configuration, the Z_2_-violation index *A(X)* was at numerical precision in all three sensing modalities. In auditory sensing, *A(X)* at ε = 0 was 1.35 × 10^∧^-16; in binocular sensing, it was 9.79 × 10^∧^-14; and in tactile sensing, it was 1.23 × 10^∧^-16. These values indicate Z_2_-equivariant Fisher-information profiles at the symmetric configuration. In Figure 2, values below 10^∧^-12 are displayed as zero so that numerical interpolation noise near machine precision is not visually mistaken for meaningful biological or computational asymmetry. The exact numerical values are retained in Table 1.

**Table 1:** Z_2_-violation index at the symmetric configuration and maximum over each sensor-asymmetry sweep

Modality	Asymmetry	*A(X)* at epsilon = 0	Maximum *A(X)*	Epsilon at maximum
Auditory	shift	1.35 × 10^∧^-16	1.35 × 10^∧^-16	flat
Auditory	tilt	1.35 × 10^∧^-16	1.273	−0.785
Auditory	front-back	1.35 × 10^∧^-16	1.273	+1.000
Binocular	shift	9.79 × 10^∧^-14	1.46 × 10^∧^-13	flat
Binocular	tilt	9.79 × 10^∧^-14	1.298	−0.785
Binocular	front-back	9.79 × 10^∧^-14	1.322	+1.000
Tactile	shift	1.23 × 10^∧^-16	1.23 × 10^∧^-16	flat
Tactile	tilt	1.23 × 10^∧^-16	4.82 × 10^∧^-16	numerical edge
Tactile	front-back	1.23 × 10^∧^-16	1.23 × 10^∧^-16	flat

The effect of geometric deformation depended on both the deformation type and the task space. Longitudinal shift moved the paired sensors together along the anteroposterior axis and preserved Z_2_ equivariance in all three modalities. Tilt rotated the sensor-pair axis relative to the anteroposterior and left-right axes, and front-back asymmetry staggered the two sensors along the anteroposterior axis. These latter two deformations violated Z_2_ equivariance in auditory and binocular sensing. In the auditory model, the maximum Z_2_ violation reached 1.273 under tilt and 1.273 under front-back asymmetry. In the binocular model, the corresponding maxima were 1.298 and 1.322. In the tactile model, all three deformations remained at numerical precision because the tactile cue was defined on a one-dimensional body surface and depended only on the x-coordinates of the receptors. The maximum values are summarized in Table 1.

These results show that bilateral symmetry produces left-right balanced local estimation precision at the sensing stage. However, they do not imply that directional sign is present in the scalar readout. Directional sign was therefore quantified separately in the processing-stage analysis.

### Processing asymmetry selectively increased sign information while reducing unsigned information

In this section, *m* denotes the scalar auditory readout obtained by weighting the cross-correlation *C*_12_(*τ*; *φ*) with the processing kernel *k(τ)*. Therefore, I(*φ*; *m*) measures information between source direction and the scalar readout, I(sign(*φ*); *m*) measures information about source side, and I(|*φ*|; *m*) measures unsigned information about angular eccentricity.

**Figure 3:**
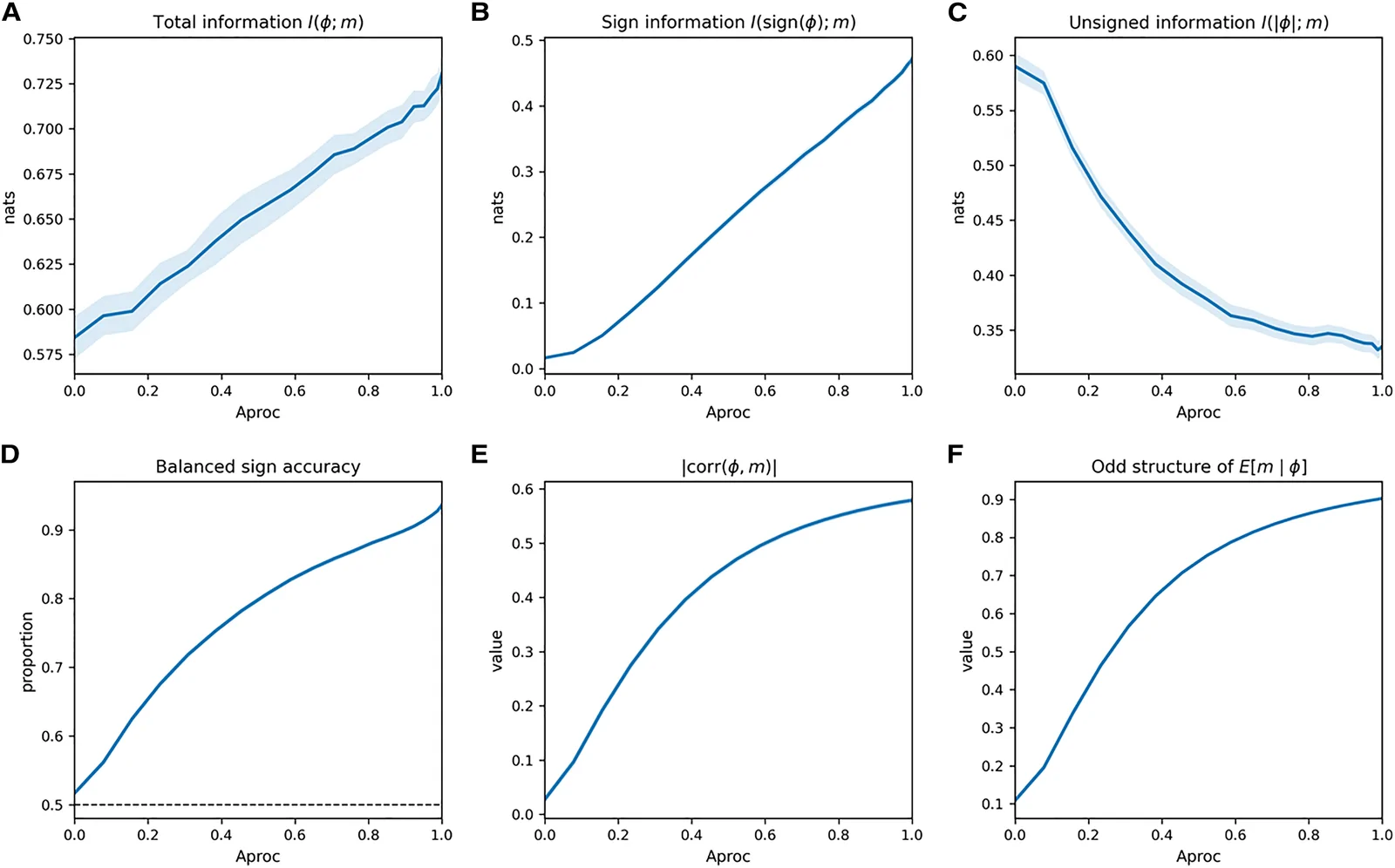
Effects of processing asymmetry on directional readout Dependence of directional readout performance on the processing-asymmetry parameter *A*proc in the auditory cross-correlation model. The scalar readout *m* was obtained by weighting the cross-correlation *C*_12_(*τ*; *φ*) with the kernel *k(τ)*. Panels show total mutual information *I*(*φ*; *m*), sign information *I*(sign(*φ*); *m*), unsigned spatial information *I*(|*φ*|; *m*), balanced sign accuracy, absolute correlation magnitude |corr(*φ*, *m*)|, and the odd structure of the conditional mean E[*m* | *φ*]. Increasing *A*proc increased directional-sign information, sign-recovery accuracy, correlation magnitude, and odd structure in the readout, while reducing unsigned spatial information. The dashed horizontal line in the balanced-sign-accuracy panel marks chance level at 0.5. Shaded regions indicate 95% confidence intervals across seeds.

At symmetric sensor placement, a purely even readout preserved substantial unsigned spatial information but did not recover directional sign. At *Aproc* = 0, total information was I(*φ*; *m*) = 0.584 ± 0.012 nats, and unsigned information was I(|*φ*|; *m*) = 0.590 ± 0.012 nats. In contrast, sign information was near zero, I(sign(*φ*); *m*) = 0.0165 ± 0.0014 nats, and balanced sign accuracy was 0.5168 ± 0.0034, close to chance (Figure 3).

Increasing *Aproc* shifted the readout from unsigned information toward directional sign information. I(sign(*φ*); *m*) rose monotonically across the sweep, reaching 0.471 ± 0.009 nats at *Aproc* = 1. Balanced sign accuracy increased to 0.9366 ± 0.0031. The polarity-invariant correlation |corr(*φ*, *m*)| increased from 0.0277 ± 0.0070 to 0.579 ± 0.006, and the odd structure of the conditional mean increased from 0.108 ± 0.0068 to 0.903 ± 0.0033 (Figure 3). By contrast, I(|*φ*|; *m*) decreased from 0.590 ± 0.012 to 0.335 ± 0.008 (Table 2). Thus, the odd component did not merely increase total information; it changed the content of the readout from unsigned spatial magnitude toward directional sign.

**Table 2:** Endpoint values for the processing-asymmetry sweep

Measure	Aproc = 0, even readout	Aproc = 1, odd readout
*I*(*φ*; *m)* [nats]	0.584 ± 0.012	0.731 ± 0.012
*I*(sign(*φ*); *m*) [nats]	0.0165 ± 0.0014	0.471 ± 0.009
*I*(|*φ*|; *m*) [nats]	0.590 ± 0.012	0.335 ± 0.008
Balanced sign accuracy	0.5168 ± 0.0034	0.9366 ± 0.0031
|corr(*φ*, *m*)|	0.0277 ± 0.0070	0.579 ± 0.006
Odd structure of E[*m* |*φ*]	0.108 ± 0.0068	0.903 ± 0.0033

Values are means ±95% confidence intervals across 50 seeds.

The separation between sign information and unsigned information was stable across histogram-bin choices. Across 10, 15, 20, 30, and 40 bins, I(sign(*φ*); *m*) remained low for the even endpoint, ranging from 0.0089 to 0.0296 nats, and remained high for the odd endpoint, ranging from 0.393 to 0.505 nats. Balanced sign accuracy was independent of the bin number because it was computed directly from sign classification, remaining 0.5168 for the even endpoint and 0.9366 for the odd endpoint across all bin choices.

### A decoder trained on the full cross-correlation acquired an odd-like readout structure

**Figure 4:**
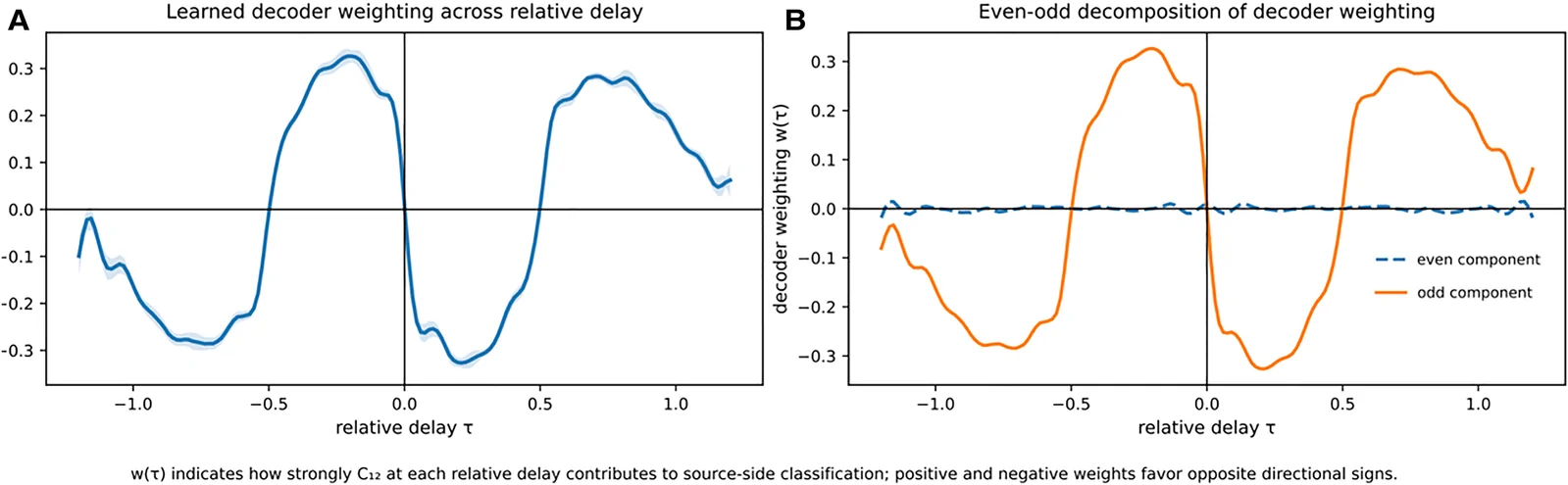
Learned full-correlation decoder and even-odd decomposition (A) Decoder weighting learned from the full cross-correlation vector as a function of relative delay *τ*. The weighting function *w*(*τ*) indicates how strongly the value of *C*_12_ at each relative delay contributes to classification of source side; positive and negative weights favor opposite source sides. (B) Even-odd decomposition of the learned weighting function. The learned decoder acquired a predominantly odd component, whereas the even component remained close to zero. This result shows that, when source side is learned directly from the full cross-correlation pattern, the inferred weighting rule is approximately antisymmetric across relative delay. Shaded regions indicate 95% confidence intervals across seeds where applicable.

The scalar-kernel analysis imposed a predefined even-to-odd family of readouts. To test whether odd structure also emerged when the readout was learned from the full cross-correlation, a logistic decoder was trained on the complete C12(*τ*) vector. The learned decoder achieved balanced sign accuracy of 0.927 ± 0.007 on held-out trials (Figure 4; Table 3). This performance was close to that of the scalar odd readout and far above the scalar even readout.

**Table 3:** Performance and weight structure of the learned full-correlation decoder

Measure	Mean ±95% confidence interval
Full-correlation decoder accuracy	0.927 ± 0.007
Full-correlation decoder balanced accuracy	0.927 ± 0.007
Odd-energy fraction of learned weight	0.994 ± 0.0016
Scalar even readout balanced accuracy	0.518 ± 0.009
Scalar odd readout balanced accuracy	0.939 ± 0.006
Scalar even readout I(sign(*φ*); m) [nats]	0.021 ± 0.004
Scalar odd readout I(sign(*φ*); m) [nats]	0.474 ± 0.020

The learned decoder weight w(*τ*) indicates how strongly the cross-correlation value at each relative delay contributes to classifying the source side. Positive and negative weights favor opposite directional signs. The learned weight profile was dominated by its odd component. The even component remained near zero across the delay axis, whereas the odd component carried almost all of the learned weight structure (Figure 4). The odd-energy fraction was 0.994 ± 0.0016. Thus, when sign classification was learned directly from the full cross-correlation, the decoder selected an almost purely odd delay-weighting pattern.

This result supports the conclusion that the odd component is not only sufficient when explicitly imposed, but is also the dominant structure selected by a decoder trained to recover directional sign from symmetric bilateral input.

### Sign recovery was robust to noise but depended on angular range and kernel scale

**Figure 5:**
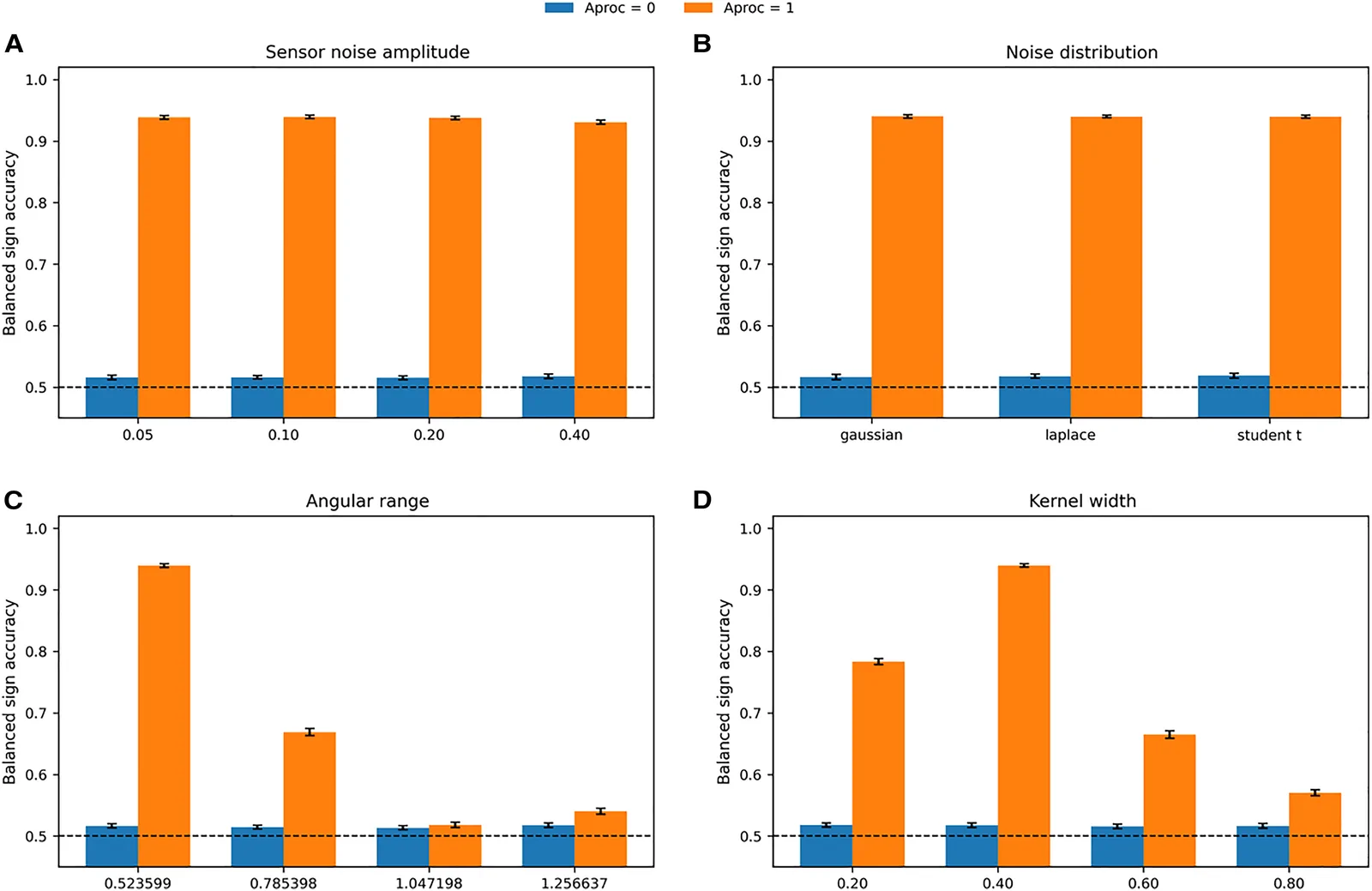
Robustness of sign recovery Endpoint robustness of sign recovery for even and odd readouts. Blue bars show the purely even endpoint, *Aproc* = 0, and orange bars show the purely odd endpoint, *Aproc* = 1. The dashed horizontal line indicates chance-level balanced sign accuracy. (A) Sign recovery remained high for the odd endpoint across sensor-noise amplitudes from 0.05 to 0.40, whereas the even endpoint remained near chance. (B) The same separation persisted under Gaussian, Laplace, and Student-t noise. (C) Increasing the angular range reduced odd-endpoint performance, indicating that the minimal odd kernel was most effective in the narrow-angle regime. (D) Kernel width also affected performance, with the strongest sign recovery at an intermediate kernel width. Error bars indicate 95% confidence intervals across seeds.

The endpoint robustness analyses compared the even endpoint, *Aproc* = 0, with the odd endpoint, *Aproc* = 1. Across sensor-noise amplitudes from 0.05 to 0.40, the even endpoint remained near chance, whereas the odd endpoint maintained high balanced sign accuracy between 0.931 and 0.939 (Figure 5A). The same qualitative pattern held across Gaussian, Laplace, and Student-t noise models: the even endpoint remained near 0.52, and the odd endpoint remained near 0.94 (Figure 5B).

The performance of the odd endpoint was not uniform across all operating regimes. Increasing the angular range reduced sign recovery: balanced sign accuracy fell from 0.939 at *φ*_max = 0.523599 to 0.669 at *φ*_max = 0.785398, and approached chance for wider ranges (Figure 5C). Kernel width also showed a matching effect. The odd endpoint performed best at sigma_*k* fraction = 0.40, with balanced sign accuracy of 0.940, but declined to 0.783 at 0.20, 0.665 at 0.60, and 0.570 at 0.80 (Figure 5D). Thus, an odd component was necessary for sign recovery under symmetric placement, but the quantitative effectiveness of the minimal odd kernel depended on the match between angular range, cross-correlation structure, and kernel scale.

Additional robustness analyses are summarized in Table 4. Asymmetric source priors did not alter the main pattern when balanced accuracy was used. Source-distribution manipulations preserved the even-versus-odd separation, although edge clustering reduced odd-endpoint performance relative to central clustering. Signal spectrum and sensor spacing exposed stronger limitations: low-band and broad-band sources supported high odd-endpoint accuracy, whereas high-band sources did not. Larger sensor spacing also reduced odd-endpoint performance.

**Table 4:** Additional robustness conditions not shown in Figure 5

Condition group	Condition value	Even endpoint balanced accuracy	Odd endpoint balanced accuracy
Source prior	symmetric	0.516	0.938
Source prior	left-heavy	0.519	0.941
Source prior	right-heavy	0.517	0.939
Source distribution	uniform	0.516	0.936
Source distribution	central cluster	0.509	0.993
Source distribution	edge cluster	0.511	0.905
Signal spectrum	baseline	0.518	0.939
Signal spectrum	low-band	0.512	0.994
Signal spectrum	high-band	0.516	0.521
Signal spectrum	broad-band	0.510	0.989
Sensor spacing	0.50	0.513	0.998
Sensor spacing	1.00	0.518	0.938
Sensor spacing	1.50	0.517	0.734
Sensor spacing	2.00	0.513	0.733

Together, these results define the operating regime of the model. Under symmetric sensor placement, even readout preserved unsigned spatial information but remained sign-blind. Odd readout produced strong directional sign recovery in the matched narrow-angle regime and remained robust to noise amplitude, noise distribution, source-prior asymmetry, and several source-distribution changes. However, high-frequency spectra, wide angular ranges, and mismatched kernel scales reduced the effectiveness of the minimal odd readout.

## Discussion

### Summary of findings

This study examined how bilaterally symmetric sensing and asymmetric processing can jointly support directional inference. The model separated the problem into two stages. The sensing stage determined how paired sensors represent directional variables under left-right reflection. The processing stage determined whether the paired sensory comparison could be readout as directional sign.

Four findings emerged. First, symmetric sensor placement produced Z_2_-equivariant Fisher-information profiles across the auditory, binocular, and tactile models. This indicates left-right balanced local estimation precision at the sensing stage, not direct recovery of directional sign. Second, under symmetric sensor placement, a purely even scalar readout preserved unsigned spatial information but remained nearly sign-blind. Third, increasing the odd component of the readout selectively increased I(sign(φ); m), balanced sign accuracy, |corr(φ, m)|, and the odd structure of E[m |φ], while unsigned information I(|φ|; m) decreased. Fourth, a decoder trained on the full cross-correlation vector acquired an almost purely odd weight structure, indicating that antisymmetric weighting is not only sufficient when imposed but is also selected by a sign-recovery task.

The resulting interpretation is conditional but precise. In a bilaterally symmetric two-sensor system with a scalar cross-correlation readout, directional sign is not supplied by symmetric sensing alone. Symmetric sensing provides a balanced representation of the two sides, whereas sign recovery requires a readout operation that distinguishes opposite delay directions.

### Bilateral symmetry as balanced sensing rather than sign recovery

Bilateral symmetry is often discussed as a morphological or developmental feature of animal organization. Its computational role, however, can be stated more narrowly. A bilaterally symmetric sensor pair can represent opposite sides of space with matched local precision. In the present model, this appeared as Z_2_ equivariance of the Fisher-information profile: I(q; X) and I(-q; X) were equal up to numerical precision at the symmetric configuration.

This result should not be interpreted as showing that sign information is already available to all downstream readouts. Fisher information is a local precision measure. Because it depends on squared sensitivity, it does not by itself distinguish whether a downstream scalar variable carries left-right sign. The present results therefore separate two issues that are often conflated. One issue is whether the sensory geometry treats the two sides with equal precision. The other is whether a readout expresses the side on which the source lies. Symmetric sensing solves the first problem, but not necessarily the second.

This distinction clarifies the role of bilateral organization. A symmetric body plan does not need to encode left-right sign at the input stage in order to be useful. Instead, it can provide a balanced sensory basis from which later processing can extract sign. This supports a division of labor between morphology and computation: body symmetry stabilizes the input geometry, while readout asymmetry supplies the operation required for directional decision.

### Even readout preserves unsigned spatial information but remains sign-blind

The processing-stage results show why the even-odd distinction matters. A purely even kernel treats positive and negative delays equivalently. Under symmetric sensor placement and a symmetric source prior, this means that opposite source directions produce the same conditional mean readout. The readout may still vary with the magnitude of the source direction, but it cannot reliably distinguish left from right.

This was directly reflected in the information measures. At the even endpoint, I(|φ|; m) remained high, whereas I(sign(φ); m) was near zero and balanced sign accuracy remained close to chance. Thus, the even readout was not uninformative. It retained unsigned spatial structure. What it lacked was directional sign.

Adding an odd component changed the informational content of the readout. I(sign(φ); m), balanced sign accuracy, |corr(φ, m)|, and odd structure all increased strongly as Aproc increased. At the same time, I(|φ|; m) decreased. This indicates that the odd component did not simply add more information to the same readout. Instead, it reorganized the readout from an unsigned representation toward a sign-sensitive representation. This is the central computational result of the study.

### Relation to biological lateralization

The coexistence of bilateral body plans and functional lateralization is widespread across animals.^1^^,^^2^^,^^3^ These two features are sometimes discussed as if they belonged to separate explanatory domains: bilateral symmetry to body plan and lateralization to neural specialization. The present model provides a way to connect them without reducing one to the other.

In this framework, a symmetric body and an asymmetric readout perform different computational roles. The symmetric sensor arrangement prevents one side of space from being locally privileged at the input stage. The asymmetric readout then converts a balanced bilateral comparison into a signed directional variable. Lateralization is therefore not simply a deviation from symmetry. Under symmetric sensing, it can be understood as a functional operation that recovers information that an even readout cannot express.

This interpretation is compatible with known principles of bilateral sensory processing. In audition, interaural time differences provide a major cue for horizontal sound localization, and downstream circuits must read the sign and magnitude of delay structure to support directional behavior.^8^^,^^9^^,^^13^ The present model does not attempt to reproduce detailed auditory circuitry. Its contribution is more abstract: it identifies the symmetry condition under which an antisymmetric readout becomes necessary for scalar sign recovery.

### Sensing-stage asymmetry defines the boundary of the claim

The model also shows when the processing-stage account no longer applies in its strict form. When sensor geometry itself breaks Z_2_ symmetry, the sensing stage can contribute directional structure before the readout. This appeared in the tilt and front-back asymmetry conditions for auditory and binocular sensing. In such cases, sign recovery need not depend only on the odd component of the processing kernel.

This boundary condition is important. The claim is not that all directional inference in biological systems must arise from processing-stage asymmetry. There are two structurally distinct routes to directional sign. In one route, the sensor geometry is bilaterally symmetric, and sign recovery requires antisymmetric processing. In the other route, the sensor geometry is itself asymmetric, and sign information can be introduced at the sensing stage. These routes can coexist, but they are not equivalent. Sensing-stage asymmetry ties directional sign to a particular body geometry, whereas processing-stage asymmetry can generate sign from a balanced bilateral input.

Biological examples of sensory asymmetry fit naturally into this distinction. In owls, for example, ear asymmetry contributes to spatial localization cues and therefore represents a case in which the sensing apparatus itself contributes directional structure.^14^^,^^15^^,^^16^ Such cases do not contradict the present model. They occupy a different region of the same sensing-processing design space.

### Learned decoding supports the functional role of odd structure

The learned-decoder analysis addressed whether odd structure was merely imposed by the predefined kernel family. When a logistic decoder was trained on the full cross-correlation vector, its learned weight was dominated by the odd component, with the even component remaining close to zero. This indicates that an antisymmetric weighting pattern is naturally selected when the task is to classify directional sign from a symmetric bilateral cross-correlation input.

This result strengthens the interpretation of the scalar-kernel analysis. The odd kernel is not only an analytically convenient construction. It corresponds to the structure that a sign decoder learns when allowed to weight the full delay axis. In this sense, odd readout structure is task-aligned: it reflects the requirement that opposite delay directions be mapped to opposite output signs.

The learned decoder also clarifies the relation between minimal and richer readouts. A scalar odd kernel is a deliberately minimal readout. A trained full-correlation decoder has more degrees of freedom. Yet both converge on the same qualitative requirement: sign recovery from symmetric bilateral delay structure depends on antisymmetric weighting.

### Robustness and operating regime

The even-versus-odd separation was robust to several changes in the model. Across sensor-noise amplitudes and across Gaussian, Laplace, and Student-t noise, the even endpoint remained near chance for sign recovery, whereas the odd endpoint maintained high balanced sign accuracy. Asymmetric source priors also did not eliminate the separation when balanced accuracy was used. These results indicate that the main effect is not an artifact of a single noise amplitude, a single Gaussian noise assumption, or a perfectly balanced sign prior.

At the same time, the quantitative success of the minimal odd kernel was regime-dependent. Increasing the angular range reduced performance, and kernel width showed a clear matching effect. The strongest sign recovery occurred when the kernel width matched the delay structure produced by the source range and sensor spacing. High-frequency signal spectra and larger sensor spacing also reduced performance. These limitations are expected for a minimal single-scale kernel. The odd component is necessary under the stated symmetry assumptions, but a particular minimal odd kernel is not guaranteed to be sufficient across all possible signal spectra, angular ranges, or geometries.

This distinction between necessity and sufficiency is central. The symmetry argument identifies why an even scalar readout is sign-blind under symmetric placement. The simulations show that a simple odd readout can restore sign recovery in a matched narrow-angle regime. They do not imply that the same single-scale odd kernel is optimal for all bilateral sensory systems. Richer biological or learned systems may combine multiple temporal scales, nonlinear transformations, adaptive weighting, or circuit-specific mechanisms.

### Implications

The results support a compact interpretation of bilateral computation. A symmetric body plan can provide balanced sensory access to opposite sides of space. However, balanced sensing is not the same as signed directional readout. To convert a symmetric bilateral comparison into a left-right decision, the processing stage must contain an operation that changes sign under the left-right reflection.

This interpretation reframes the relationship between body symmetry and functional lateralization. They are not alternative explanations for directional behavior. They can be complementary components of a single inference pipeline. Symmetric sensing provides locally unbiased input, and asymmetric processing extracts directional sign from that input. Under this view, lateralization is not merely an exception to bilateral symmetry, but a computationally defined operation that becomes necessary when a symmetric body must produce signed directional behavior.

The broader implication is that morphology and computation should be analyzed together. A body plan determines what comparisons are available; a readout determines which aspects of those comparisons become behaviorally meaningful. In the minimal bilateral system studied here, the division is clear: symmetry at the sensing stage preserves balanced precision, and symmetry breaking at the readout stage recovers directional sign.

### Limitations of the study

The model is intentionally minimal. It uses two point sensors, simplified sensory cues, a scalar cross-correlation readout, and reduced task geometries. Real sensory systems operate in three-dimensional environments, contain multiple receptor populations, use nonstationary signals, and combine information across time, frequency, and behavior. The tactile model is especially reduced because it is defined on a one-dimensional body surface and depends only on receptor x-coordinates. As a result, y axis deformations do not affect the tactile cue in the present formulation.

The processing-stage theorem also applies to a specified class of readouts. It states that, under symmetric placement and an even scalar kernel, the conditional mean readout is sign-blind. It does not rule out sign recovery by other readout architectures when they use information not captured by the scalar even-kernel readout. The learned-decoder analysis partially addresses this issue by showing that a full-correlation decoder trained for sign classification develops an odd-like structure. However, future models should examine broader decoder classes, including nonlinear decoders, recurrent circuits, adaptive kernels, and embodied sensorimotor feedback.

Another limitation is that the present analysis treats left-right reflection as the relevant symmetry. Many biological systems face richer symmetry structures, including three-dimensional rotations, front-back ambiguities, vertical localization, active sensing movements, and context-dependent priors. Extending the framework to these cases would allow one to test whether analogous decompositions appear for other symmetry groups and other forms of directional decision.

## Resource availability

### Lead contact

Further information and requests should be directed to the lead contact, Nobuchika Yamaki (nobuchika.yamaki@tnqtech.ooo).

### Materials availability

This study did not generate new unique reagents or biological materials.

### Data and code availability

All simulation code, analysis scripts, and numerical data generated in this study are publicly available on Zenodo at https://doi.org/10.5281/zenodo.19648083.

## Acknowledgments

This study received no external funding.

## Author contributions

N.Y designed the study, performed all analyses, drafted the manuscript, and approved the final version. C.T developed the simulation code and contributed to the implementation of the computational framework.

## Declaration of interests

Authors declare no conflicts of interest related to this work.

## STAR★Methods

### Key resources table

**Table:** 

REAGENT or RESOURCE	SOURCE	IDENTIFIER
**Deposited data**		

Simulation code, analysis scripts, and numerical outputs	This paper	Zenodo: https://doi.org/10.5281/zenodo.19648083

**Software and algorithms**		

Python 3.13.2	Python Software Foundation	https://www.python.org/
NumPy 2.4.2	NumPy Developers	https://numpy.org/
Matplotlib 3.10.8	Matplotlib Development Team	https://matplotlib.org/

### Method details

We analyzed a minimal bilateral inference system with two sequential stages. The first stage was a sensing stage, in which two spatially separated sensors received signals from an external source. The second stage was a processing stage, in which the paired sensor signals were combined into either a scalar readout or a learned decoder.

The directional variable was denoted by *φ*. In the auditory and binocular models, *φ* represented source direction in the horizontal plane, viewed from the dorsal side of the body. The y-axis was defined as the anteroposterior axis, with positive y pointing anteriorly. The x-axis was defined as the left-right axis. *φ* was measured from the positive anteroposterior axis toward the positive left-right side. Thus, positive *φ* denoted a source on the positive left-right side of the body midline, and negative *φ* denoted the mirror-symmetric source on the opposite side. The directional sign was sign(*φ*), which indicates source side, and the unsigned directional magnitude was |*φ*|, which indicates angular eccentricity from the midline without specifying side. In the tactile model, the task variable was x_p_, the position of a stimulus on a one-dimensional body surface along the left-right axis.

The left-right reflection was the operation that exchanges the two sides of the body. For the auditory and binocular models, this reflection maps *ϕ* to −*ϕ*. For the tactile model, it maps *x*_p_ to −*x*_p_. This reflection was denoted as Z_2_ because applying the operation twice returns the original state.

The relative-delay axis *τ* was used only in the auditory processing-stage model. The auditory model contains a natural temporal comparison because the same source signal reaches the two sensors with different arrival times depending on source side. The binocular and tactile models were used for sensing-stage Fisher-information analyses only. They were not assigned temporal-delay readouts, and no *τ*-based cross-correlation analysis was applied to the binocular or tactile models.

Fisher information was used to quantify local estimation precision at the sensing stage. It was not treated as a direct measure of directional sign information. Directional sign recovery at the processing stage was quantified separately using I(sign(*ϕ*); *m*), I(|*ϕ*|; *m*), sign-recovery accuracy, balanced sign-recovery accuracy, and learned-decoder performance.

#### Sensor geometry

Each model contained two point sensors in a two-dimensional dorsal-view body plane. The y-axis represented the anteroposterior axis, and the x-axis represented the left-right axis. The baseline bilaterally symmetric configuration placed the two sensors at equal distances from the body midline along the left-right axis. This geometry corresponds to a paired bilateral sensory arrangement, such as two ears, two eyes, or two tactile receptor positions.

The baseline bilaterally symmetric configuration was

(Equation 1) $$x_{1}=\left(-d\;/\;2,0\right),x_{2}=\left(+d\;/\;2,0\right)\text{.}$$

Here, x_1_ and x_2_ denote the two sensor positions, d is the distance between the sensors, the x-axis is the left-right axis, and the positive y-axis is the anterior direction along the anteroposterior axis. Unless otherwise stated, d = 1.

Three geometric deformations were applied to the baseline bilateral sensor pair.

The first deformation was a longitudinal shift along the anteroposterior axis. In biological geometric terms, this moves the entire paired sensor arrangement anteriorly or posteriorly without changing the left-right relation between the two sensors:

(Equation 2) $${x_{1}}^{\prime }=x_{1}+\left(0,\varepsilon shift\right),{x_{2}}^{\prime }=x_{2}+\left(0,\varepsilon shift\right)$$

This deformation preserves left-right reflection symmetry because both sensors are shifted equally along the anteroposterior axis.

The second deformation was a tilt of the bilateral sensor-pair axis relative to the left-right and anteroposterior body axes. This deformation rotates the line connecting the two sensors around the origin:

(Equation 3) R(εtilt)=[[cos(εtilt),−sin(εtilt)],[sin(εtilt),cos(εtilt)]].

The tilted sensor positions were

(Equation 4) $${x_{1}}^{\prime }=R\left(\varepsilon tilt\right)\;x_{1},{x_{2}}^{\prime }=R\left(\varepsilon tilt\right)\;x_{2}\text{.}$$

A generic nonzero value of εtilt makes the sensor-pair axis oblique relative to the body axes and breaks reflection symmetry about the original anteroposterior axis.

The third deformation was a front-back asymmetry. In biological geometric terms, this produces a staggered bilateral pair, in which one sensor is displaced posteriorly and the other anteriorly:

(Equation 5) $${x_{1}}^{\prime }=x_{1}+\left(0,-\varepsilon front-back\;/\;2\right)\text{,}$$

(Equation 6) $$xˆ2^{\prime }=xˆ2+\left(0,+\varepsilon front-back\;/\;2\right)\text{.}$$

This deformation introduces sensor-level handedness because the two sensors are no longer mirror images with respect to the original anteroposterior axis.

#### Auditory sensing model

For auditory horizontal localization, the source direction vector was

(Equation 7) u(φ)=(sin(φ),cos(φ)).

The baseline vector between the two sensors was

(Equation 8) $$\Delta =x_{2}-x_{1}\text{.}$$

The noise-free interaural-time-difference cue was

(Equation 9) μauditory(φ;X)=Δdotu(φ)/c.

Here, *X* denotes the sensor configuration and *c* is the propagation speed. The noisy observation was

(Equation 10) s=μauditory(φ;X)+η,

where *η* was zero-mean Gaussian observation noise with variance *σ*^2^. The derivative of the direction vector was

(Equation 11) du(φ)/dφ=(cos(φ),−sin(φ)).

The auditory Fisher information was

(Equation 12) $$Iauditory\left(\varphi ;\;X\right)={\left[\Delta \;dot\;\left(du\left(\varphi \right)\;/\;d\varphi \right)\;/\;c\right]}^{2}\;/\;\sigma^{2}\text{.}$$

For the sensing-stage analysis, *ϕ* was sampled over [−π, π) using 721 grid points.

#### Binocular sensing model

For binocular localization, the source was placed at fixed reference distance *R* from the midpoint:

(Equation 13) r(φ)=(Rsin(φ),Rcos(φ)).

The viewing angle from sensor *i* to the source was

(Equation 14) $$\alpha ᵢ\left(\varphi \right)=\mathrm{atan}\;2\left(r_{x}\left(\varphi \right)-x{ᵢ,}_{x},rᵧ\left(\varphi \right)-xᵢ,ᵧ\right)\text{.}$$

The binocular disparity cue was

(Equation 15) $$\mu binocular\left(\varphi ;\;X\right)=\alpha_{1}\left(\varphi \right)-\alpha_{2}\left(\varphi \right)\text{.}$$

The noisy observation was

(Equation 16) s=μbinocular(φ;X)+η,

where *η* had variance *σ*^2^. The binocular Fisher information was

(Equation 17) $$Ibinocular\left(\varphi ;\;X\right)={\left[d\mu binocular\left(\varphi ;\;X\right)\;/\;d\varphi \right]}^{2}\;/\;\sigma^{2}\text{.}$$

The derivative *dμ*binocular/*dϕ* was evaluated numerically on the sampled *ϕ* grid. The binocular source direction was sampled over [−π/2 + 0.001, π/2–0.001] using 721 grid points. The reference distance was *R* = 5.

#### Tactile sensing model

For tactile localization, the stimulus coordinate was *x*_p_ on a one-dimensional body surface. Each sensor had a Gaussian receptive field centered at the *x*-coordinate of that sensor:

(Equation 18) $$G\left(u\right)=\exp\left[-u^{2}\;/\;\left(2l^{2}\right)\right]\text{.}$$

The tactile differential cue was

(Equation 19) $$\mu tactile\left(x_{p};\;X\right)=G\left(x_{p}-x_{1}{,}_{x}\right)-G\left(x_{p}-x_{2}{,}_{x}\right)\text{.}$$

The noisy observation was

(Equation 20) $$s=\mu tactile\left(x_{p};\;X\right)+\eta \text{,}$$

where *η* had variance *σ*^2^. The derivative of the tactile cue was

(Equation 21) $$d\mu tactile\left(x_{p};\;X\right)/{dx}_{p}=-\left[\left(x_{p}-x_{1}{,}_{x}\right)/\;l^{2}\right]\;G\left(x_{p}-x_{1}{,}_{x}\right)+\left[\left(x_{p}-x_{2}{,}_{x}\right)/l^{2}\right]\;G\left(x_{p}-x_{2}{,}_{x}\right)\text{.}$$

The tactile Fisher information was

(Equation 22) $$Itactile\left(x_{p};\;X\right)={\left[d\mu tactile\left(x_{p};\;X\right)\;/\;{dx}_{p}\right]}^{2}\;/\;\sigma^{2}\text{.}$$

The tactile coordinate *x*_p_ was sampled over [−2, 2] using 721 grid points, and l = 0.5. Because this tactile model is one-dimensional and depends only on the *x*-coordinates of the sensors, deformations that only alter the *y*-coordinates do not alter the tactile cue.

#### Z_2_ equivariance of Fisher-information

For each modality, the Fisher-information profile was tested for equivariance under the left-right reflection. The task variable was denoted by *q*, where *q* = *ϕ* for auditory and binocular localization and *q* = *x*_p_ for tactile localization. The Fisher-information profile was Z_2_-equivariant when

(Equation 23) I(q;X)=I(−q;X)forallsampledq.

The normalized Z_2_-violation index was

(Equation 24) A(X)=meanoverq|I(q;X)−I(−q;X)|/meanoverqI(q;X).

Here, mean over *q* denotes the arithmetic mean over the sampled task grid. Auditory direction was treated as a periodic angular variable, so (−*q*; *X*) was evaluated by periodic interpolation. Binocular direction and tactile body position were treated as non-periodic variables, so (−*q*; *X*) was evaluated by non-periodic interpolation within the sampled domain. (*X*) = 0 indicates exact Z_2_ equivariance up to numerical precision, whereas *A*(*X*) > 0 indicates violation of left-right equivariance in the Fisher-information profile.

For the sensing-stage sweeps, *ε*shift and *ε*front-back were sampled over [−1, 1] using 41 grid points. *ε*tilt was sampled over [−π/2, π/2] using 41 grid points. The observation-noise standard deviation was *σ* = 0.05. The deformation ranges in this sensing-stage analysis were chosen to visualize the symmetry properties of the Fisher-information profiles over a broad geometric range. They are distinct from the narrower joint geometry-processing sweeps described under Joint geometry-processing sweeps.

#### Auditory processing-stage signal model

The processing-stage cross-correlation analyses used only the auditory model. This is because the auditory model contains a natural temporal comparison: the same source signal reaches the two sensors with different arrival times depending on source side. This temporal difference is the model analogue of an interaural time difference. The binocular and tactile models were not given temporal-delay readouts; they were used only to test sensing-stage Z_2_ equivariance of Fisher-information profiles.

The two sensor signals were generated from a common source signal (*t*) with sensor-specific delays and additive sensor noise:

(Equation 25) $$s_{1}\left(t\right)=x\left(t-\tau_{1}\left(\varphi \right)\right)+\eta_{1}\left(t\right)\text{,}$$

(Equation 26) $$s_{2}\left(t\right)=x\left(t-\tau_{2}\left(\varphi \right)\right)+\eta_{2}\left(t\right)\text{.}$$

The delay at sensor *i* was

(Equation 27) τᵢ(φ)=xᵢdotu(φ)/c.

Fractional delays were implemented by linear interpolation. *η*_1_(*t*) and *η*_2_(*t*) were independent noise processes with the same scale parameter.

The source signal (*t*) was generated as bandpass-filtered white noise. A white-noise time series was transformed into the frequency domain, frequencies outside the selected band were set to zero, and the signal was transformed back into the time domain. The resulting signal was normalized to unit standard deviation. The default frequency band was [1, 8] in normalized frequency units. The default sampling frequency was *f*s = 2000, and the signal duration was *T*sig = 2.0.

The default source-direction prior was uniform over [−*ϕ*max, +*ϕ*max], with *ϕ*max = π/6. The default sensor-noise model was independent Gaussian noise with standard deviation *σ*noise = 0.20.

#### Cross-correlation and scalar readout

For each auditory trial, the cross-correlation between the two sensor signals was computed as

(Equation 28) $$C_{12}\left(\tau ;\;\varphi \right)=\left(1\;/\;T\right)\int s_{1}\left(t\right)\;s_{2}\left(t-\tau \right)\;dt\text{.}$$

*C*_12_(*τ*; *φ*) measures how well the signal at sensor *x*_1_ matches the signal at sensor *x*_2_ after shifting one signal relative to the other by delay *τ*. The subscript 12 indicates that the comparison is between sensors *x*_1_ and *x*_2_. Under the convention in Equation 28, positive *τ* denotes the relative-delay direction in which the signal at *x*_2_ is delayed relative to the signal at *x*_1_, whereas negative *τ* denotes the opposite delay direction. Thus, positive and negative values of *τ* correspond to opposite arrival-time orders across the bilateral sensor pair. In this study, the *τ*-based cross-correlation readout was used only for the auditory processing-stage model.

*C*_12_(*τ*; *φ*) was evaluated on a uniform delay grid over [-*τ*max, +*τ*max]. The maximum sampled delay was

(Equation 29) τmax=1.2d/c.

The number of sampled delay points was 161. The scalar readout for a kernel *k*(*τ*) was

(Equation 30) $$m_{k}\left(\varphi \right)=\int k\left(\tau \right)\;C_{12}\left(\tau ;\;\varphi \right)\;d\tau \text{.}$$

Here, *m*_k_(*φ*), abbreviated as *m* in the information-theoretic analyses, denotes the single scalar output produced by reading the auditory cross-correlation with the kernel *k*(*τ*). Thus, *I*(*φ*; *m*), *I*(sign(*φ*); *m*), and *I*(|*φ*|; *m*) quantify how much the scalar readout *m* contains about total source direction, source side, and unsigned source eccentricity, respectively. Numerically, the integral in Equation 30 was approximated by a Riemann sum over the sampled *τ* grid.

#### Even and odd readout kernels

A readout kernel *k(τ)* is the set of weights applied to the cross-correlation values at different relative delays. In biological terms, it specifies which delay relationships between the two sensors are emphasized when the paired sensory signals are converted into a single readout variable. A positive weight at a given *τ* increases the readout when *C*_12_ is large at that delay, whereas a negative weight decreases the readout.

Any readout kernel *k(τ)* was decomposed into an even component and an odd component:

(Equation 31) keven(τ)=[k(τ)+k(−τ)]/2,

(Equation 32) kodd(τ)=[k(τ)−k(−τ)]/2.

An even kernel treats positive and negative relative delays equivalently. It therefore reads the strength of the bilateral match without distinguishing which side led in time. An odd kernel assigns opposite signs to opposite relative delays. It can therefore distinguish left-leading from right-leading delay structure. Even and odd kernels are not merely alternative names for symmetry and asymmetry; they define two different readout operations on the same paired sensory comparison. The default integral-kernel family was

(Equation 33) k(τ;γ)=cos(γ)keven,base(τ)+sin(γ)kodd,base(τ),

where

(Equation 34) $$keven,base\left(\tau \right)=\exp\left[-\tau^{2}\;/\;\left(2\sigma k^{2}\right)\right]\text{,}$$

(Equation 35) $$kodd,base\left(\tau \right)=\left(\tau \;/\;\sigma k\right)\;\exp\left[-\tau^{2}\;/\;\left(2\sigma k^{2}\right)\right]\text{.}$$

Each basis kernel was normalized by its maximum absolute value. The kernel-width parameter was

(Equation 36) σk=σk,fracd/c.

The default value was *σ*k,frac = 0.4. The processing-asymmetry parameter was

(Equation 37) Aproc=sin(γ).

The parameter γ controls the mixture between the even and odd kernel components. The derived index *Aproc* = sin(γ) was used as the processing-asymmetry index. *Aproc* = 0 denotes a purely even readout, which treats the two delay directions equivalently. *Aproc* = 1 denotes a purely odd readout, which assigns opposite signs to the two delay directions. Intermediate values represent graded mixtures of even and odd readout structure. Thus, *A*proc = 0 corresponds to purely even processing, and *A*proc = 1 corresponds to purely odd processing. The processing sweep used 21 uniformly spaced values of *γ* over [0, π/2].

An illustrative two-tap kernel was also defined as

$$ktwo-tap\left(\tau ;\;\gamma \right)=\cos\left(\gamma \right)\;\left[\delta \left(\tau -d_{0}\right)+\delta \left(\tau +d_{0}\right)\right]$$

(Equation 38) $$\sin\left(\gamma \right)\;\left[\delta \left(\tau -d_{0}\right)-\delta \left(\tau +d_{0}\right)\right]\text{.}$$

The tap position was

(Equation 39) $$d_{0}=0.5\;d\;/\;c\text{.}$$

The two-tap kernel was used only as an interpretable special case. Quantitative results were based on the integral-kernel family.

#### Sign-blindness of an even scalar readout

The central analytical statement applies to the scalar readout in Equation 30. It assumes bilaterally symmetric sensor placement, a symmetric source prior, stationary source statistics, independent identically distributed sensor noise, and an even readout kernel.

Under bilaterally symmetric placement, reflection of the source direction reverses the cross-correlation delay axis in expectation:

(Equation 40) $$E\left[C_{12}\left(\tau ;\;\varphi \right)\right]=E\left[C_{12}\left(-\tau ;-\varphi \right)\right]\text{.}$$

If the kernel is even, then

(Equation 41) k(τ)=k(−τ).

The conditional mean readout is

(Equation 42) $$E\left[m_{k}\;|\;\varphi \right]=\int k\left(\tau \right)\;E\left[C_{12}\left(\tau ;\;\varphi \right)\right]\;d\tau \text{.}$$

Substituting Equation 40 into Equation 42 gives

(Equation 43) $$E\left[m_{k}\;|\;\varphi \right]=\int k\left(\tau \right)\;E\left[C_{12}\left(-\tau ;-\varphi \right)\right]\;d\tau \text{.}$$

Using the change of variable *τ*′ = −*τ* gives

(Equation 44) $$E\left[m_{k}\;|\;\varphi \right]=\int k\left({-\tau }^{\prime }\right)\;E\left[C_{12}\left(\tau^{\prime };-\varphi \right)\right]\;d\tau^{\prime }\text{.}$$

Because (−*τ*′) = *k*(*τ*′), this becomes

(Equation 45) $$E\left[m_{k}\;|\;\varphi \right]=\int k\left(\tau^{\prime }\right)\;E\left[C_{12}\left(\tau^{\prime };-\varphi \right)\right]\;d\tau^{\prime }\text{.}$$

Therefore,

(Equation 46) $$E\left[m_{k}\;|\;\varphi \right]=E\left[m_{k}\;|-\varphi \right]\text{.}$$

Equation 46 states that an even scalar readout has the same conditional mean for opposite source directions under bilaterally symmetric sensing. Such a readout can preserve information about |*ϕ*| but cannot express left-right sign through its conditional mean. This statement applies to the specified scalar readout and does not exclude sign recovery by different readout classes or by sensor geometries that already break left-right symmetry.

### Quantification and statistical analysis

#### Processing-stage simulations

For each trial, *ϕ* was sampled from the specified source-direction prior, a bandpass source signal was generated, delayed sensor signals were produced, cross-correlation was computed, and the scalar readout *m*_k_ was evaluated.

The default processing-stage configuration was:

(Equation 47) d=1,c=1,fs=2000,Tsig=2.0,flo=1,fhi=8,σnoise=0.20,σk,frac=0.4,φmax=π/6,ntrials=600.

The main processing sweep was repeated across 50 random seeds. The seed for repetition *r* was

(Equation 48) seedᵣ=seedbase+r,

with seedbase = 20260418 and *r* = 0, 1, …, 49. For each value of *A*proc, the reported mean, standard deviation, and 95% confidence interval were computed across seeds.

The 95% confidence interval was

(Equation 49) CI95=mean±1.96SD/sqrt(nseeds),

where *n*seeds = 50.

#### Information-theoretic diagnostics

Mutual information was estimated by discretizing the variables into histograms. For two variables *A* and *B*, the mutual information estimate was

(Equation 50) I(A;B)=∑overa,bp(a,b)log[p(a,b)/(p(a)p(b))].

The logarithm was natural, so mutual information was reported in nats.

Three mutual-information quantities were computed:

(Equation 51) I(φ;m),

(Equation 52) I(sign(φ);m),

(Equation 53) I(|φ|;m).

(*ϕ*; *m*) measures total information between source direction and readout. (sign(*ϕ*); *m*) measures information about left-right side. (|*ϕ*|; *m*) measures unsigned spatial information. The default number of histogram bins was 20. Bin sensitivity was tested using 10, 15, 20, 30, and 40 bins.

#### Sign-recovery diagnostics

Raw sign-recovery accuracy was computed after aligning the arbitrary global polarity of the scalar readout. For nonzero *ϕ*, the two possible readout polarities were evaluated:

(Equation 54) Accuracy+=mean1[sign(m)=sign(φ)],

(Equation 55) Accuracy−=mean1[−sign(m)=sign(φ)].

The sign-recovery accuracy was

(Equation 56) Accuracy=max(Accuracy+,Accuracy−).

For asymmetric priors, balanced sign-recovery accuracy was used. Sensitivity and specificity were

(Equation 57) Sensitivity=P(predictedsign=+1|truesign=+1),

(Equation 58) Specificity=P(predictedsign=−1|truesign=−1).

Balanced sign-recovery accuracy was

(Equation 59) Balancedaccuracy=(Sensitivity+Specificity)/2.

As with raw accuracy, the global polarity of the readout was chosen to maximize balanced accuracy. Chance-level performance is 0.5.

Pearson correlation between *ϕ* and *m* was also reported:

(Equation 60) corr(φ,m)=cov(φ,m)/[SD(φ)SD(m)].

Because the global polarity of the kernel is arbitrary, the sign of corr(*ϕ*, *m*) was not interpreted as performance loss. The polarity-invariant magnitude |corr(*ϕ*, *m*)| was also reported.

#### Conditional-mean odd structure

The conditional mean of the readout was estimated by binning trials according to *ϕ*. Let (*ϕ*b) denote the mean readout in bin *b* centered at *ϕ*b. The odd component of the binned conditional mean was

(Equation 61) Modd(φb)=[M(φb)−M(−φb)]/2.

The even component was

(Equation 62) Meven(φb)=[M(φb)+M(−φb)]/2.

The normalized odd-structure index was

(Equation 63) Oddstructure=meanoverb|Modd(φb)|/SDoverbM(φb).

This quantity is near zero when the conditional mean is even in *ϕ* and increases when the readout acquires sign-sensitive odd structure.

#### Robustness analyses

Endpoint robustness was evaluated by comparing *A*proc = 0 and *A*proc = 1 under changes to model assumptions and operating regimes. Each endpoint analysis used the same trial structure and the same seed-repetition procedure as the main sweep unless otherwise specified.

The following parameter groups were tested.

Sensor-noise amplitude:

(Equation 64) σnoise∈{0.05,0.10,0.20,0.40}.

Kernel width:

(Equation 65) σk,frac∈{0.2,0.4,0.6,0.8}.

Source-angle range:

(Equation 66) φmax∈{π/6,π/4,π/3,0.4π}.

Source-direction prior:

(Equation 67) p(φ)=symmetricuniformprior,biasedprior.

For the symmetric uniform prior, *ϕ* was sampled uniformly over [−*ϕ*max, +*ϕ*max]. For the biased prior, the sampling probability of the two signs was unequal while keeping the same absolute angular support. Balanced sign-recovery accuracy was used for prior-biased conditions.

Noise model:

(Equation 68) η∼Gaussian,Laplace,Student−t.

The noise scale was matched across noise families by the specified *σ*noise parameter.

Signal spectrum:

(Equation 69) spectrum∈{low−band,baseline,broad−band,high−band}.

The baseline spectrum used the frequency band [1, 8]. The other spectrum conditions changed the retained frequency range while preserving the same source-signal generation procedure.

Source distribution:

(Equation 70) distribution∈{uniform,clustered}.

The uniform condition sampled *ϕ* uniformly over the specified angular range. The clustered condition concentrated source directions within the same angular support.

Sensor spacing:

(Equation 71) d∈{0.5,1.0,1.5,2.0}.

For each robustness condition, the endpoint results were reported for (sign(*ϕ*); *m*), *I*(|*ϕ*|; *m*), sign-recovery accuracy, balanced sign-recovery accuracy, |corr(*ϕ*, *m*)|, and odd structure.

#### Joint geometry-processing sweeps

This analysis used narrower deformation ranges than the sensing-stage Fisher-information sweep in the Z_2_ equivariance of Fisher information analysis. The purpose of the sensing-stage sweep was to evaluate whether each geometric deformation preserved or violated Z_2_ equivariance over a broad range. The purpose of the joint geometry-processing sweep was different: it tested how small-to-moderate geometric deviations around the symmetric configuration interacted with processing-stage asymmetry. Therefore, the two analyses used different ε ranges and should not be interpreted as conflicting parameter definitions.

To examine cases in which the sensing stage itself breaks left-right symmetry, each geometric deformation was combined with the processing-asymmetry sweep. For each deformation type, *ε* was sampled over 9 values and *γ* was sampled over 21 values.

For longitudinal shift:

(Equation 72) εshift∈[−0.5,+0.5].

For tilt:

(Equation 73) εtilt∈[−π/6,+π/6].

For front-back asymmetry:

(Equation 74) εfront−back∈[−0.5,+0.5].

For each pair of *ε* and *A*proc, the scalar readout was simulated and sign-recovery diagnostics were computed. These sweeps determine whether directional sign arises only at the processing stage or whether the sensor geometry itself contributes directional structure.

#### Learned full cross-correlation decoder

A learned decoder was used to test whether sign classification from the full cross-correlation vector naturally used odd-like delay structure. For each trial, the feature vector was

(Equation 75) $$c=\left[C_{12}\left(\tau_{1}\right),C_{12}\left(\tau_{2}\right),...,C_{12}\left(\tau J\right)\right]\text{,}$$

where *J* = 161 is the number of sampled delay points. The binary target was

(Equation 76) y=1ifφ>0,andy=0ifφ<0.

A logistic decoder was trained:

(Equation 77) p(y=1|c)=1/[1+exp(−(wdotc+b))].

The loss function was binary cross-entropy with L2 regularization:

(Equation 78) $$L=-mean\left[y\;\log\;p+\left(1-y\right)\;\log\left(1-p\right)\right]+\lambda \;{\left\|w\right\|}^{2}\text{.}$$

The regularization strength was *λ* = 0.01. The decoder was trained by gradient descent with learning rate 0.25 for 500 iterations. Each decoder run used 1500 trials. The training fraction was 0.7 and the test fraction was 0.3. The learned-decoder analysis was repeated across 10 random seeds.

The learned decoder weight w(*τ*) indicates how strongly the cross-correlation value at each relative delay *τ* contributes to classifying the source side. A positive weight means that a high value of *C*_12_ at that delay favors one directional sign, whereas a negative weight favors the opposite directional sign. Therefore, the shape of w(*τ*) can be interpreted as the delay-weighting strategy learned by the classifier. The learned weight vector (*τ*) was decomposed into even and odd components:

(Equation 79) weven(τ)=[w(τ)+w(−τ)]/2,

(Equation 80) wodd(τ)=[w(τ)−w(−τ)]/2.

The odd-energy fraction was

(Equation 81) $$Odd-energy\;fraction={\sum over\;\tau \;wodd\left(\tau \right)}^{2}\;/\;\left[{\sum over\;\tau \;weven\left(\tau \right)}^{2}+{\sum over\;\tau \;wodd\left(\tau \right)}^{2}\right]\text{.}$$

A value near 1 indicates that the learned sign decoder relies primarily on odd delay structure. Decoder performance was evaluated using balanced sign-recovery accuracy on the held-out test set.

#### Numerical implementation

All simulations were implemented in Python 3 using NumPy for numerical computation and Matplotlib for visualization. Random numbers were generated with NumPy default_rng. The base seed was 20260418. The sensing-stage analysis used deterministic parameter sweeps. The processing-stage scalar-readout analysis used 50 random seeds. The learned-decoder analysis used 10 random seeds.

All reported summary values were computed from saved trial-level or seed-level outputs. For multi-seed scalar-readout analyses, means, standard deviations, and 95% confidence intervals were computed across seeds. Mutual information was reported in nats. Accuracy values were reported as proportions.
